# Targeting the myeloid microenvironment in neuroblastoma

**DOI:** 10.1186/s13046-023-02913-9

**Published:** 2023-12-13

**Authors:** Marjolein C. Stip, Loes Teeuwen, Miranda P. Dierselhuis, Jeanette H. W. Leusen, Daniëlle Krijgsman

**Affiliations:** 1 Center for Translational Immunology, University Medical Center Utrecht, 3584 CX Utrecht, The Netherlands; 2 Princess Máxima Center for Pediatric Oncology, 3584 CS Utrecht, The Netherlands; 3 Center for Molecular Medicine, University Medical Center Utrecht, 3584 CX Utrecht, the Netherlands

**Keywords:** Neuroblastoma, Myeloid cells, Macrophages, Monocytes, Neutrophils, Myeloid-derived suppressor cell, Immunotherapy, Immunosuppression

## Abstract

Myeloid cells (granulocytes and monocytes/macrophages) play an important role in neuroblastoma. By inducing a complex immunosuppressive network, myeloid cells pose a challenge for the adaptive immune system to eliminate tumor cells, especially in high-risk neuroblastoma. This review first summarizes the pro- and anti-tumorigenic functions of myeloid cells, including granulocytes, monocytes, macrophages, and myeloid-derived suppressor cells (MDSC) during the development and progression of neuroblastoma. Secondly, we discuss how myeloid cells are engaged in the current treatment regimen and explore novel strategies to target these cells in neuroblastoma. These strategies include: (1) engaging myeloid cells as effector cells, (2) ablating myeloid cells or blocking the recruitment of myeloid cells to the tumor microenvironment and (3) reprogramming myeloid cells. Here we describe that despite their immunosuppressive traits, tumor-associated myeloid cells can still be engaged as effector cells, which is clear in anti-GD2 immunotherapy. However, their full potential is not yet reached, and myeloid cell engagement can be enhanced, for example by targeting the CD47/SIRPα axis. Though depletion of myeloid cells or blocking myeloid cell infiltration has been proven effective, this strategy also depletes possible effector cells for immunotherapy from the tumor microenvironment. Therefore, reprogramming of suppressive myeloid cells might be the optimal strategy, which reverses immunosuppressive traits, preserves myeloid cells as effectors of immunotherapy, and subsequently reactivates tumor-infiltrating T cells.

## Introduction

Neuroblastoma is a pediatric tumor originating from sympathoadrenal lineage dysregulation [1] which exhibits high heterogeneity in disease severity. While low- and intermediate-risk neuroblastoma patients achieve a 80–95% 5-year overall survival rate, high-risk cases only reach 45% [2]. Approximately half of the diagnosed neuroblastoma cases are classified as high-risk, with 20–30% featuring *MYCN* amplification [3]. MYCN, a proto-oncogenic transcription factor, fosters tumor cell proliferation, angiogenesis, and metastasis, concurrently suppressing immune activation [4]. In low-risk cases, no treatment or solely surgery is often curative [5, 6], while high-risk patients undergo induction chemotherapy, surgery, high-dose chemotherapy with autologous stem cell transplantation (ASCT), and radiation therapy [7]. Since 2015, anti-disialoganglioside (GD2) antibody immunotherapy and *13-cis* retinoic acid (isotretinoin) is applied as consolidation therapy. Initially anti-GD2 was combined with IL-2 and Granulocyte/Macrophage-Colony Stimulating Factor (GM-CSF) (USA) or IL-2 only (Europe). Since the administration of IL-2 has been proven to be of no additional benefit this has been omitted [8].

Neuroblastoma has a low mutational burden [9] as well as low human leukocyte antigen (HLA) type I expression [10], limiting tumor recognition by the T cells. Due to the scarcity of neo-antigens, the development of immunotherapeutics, including anti-tumor antibodies, T cell vaccines and chimeric antigen receptor (CAR) T cells hampered initially. However, success was achieved by targeting the highly expressed GD2 [11] using antibody therapy, thereby activating NK cells and myeloid cells. Anti-GD2 antibody therapy significantly improved survival for high-risk neuroblastoma patients [12, 13]. Although GD2-targeting CAR T cells have been developed, they failed to enhance survival initially, likely due to T cell exhaustion by the immunosuppressive tumor microenvironment (TME) (reviewed in [13]). However, recently, a phase I/II trial showed that the use of GD2-targeting CAR T cells (GD2-CART01) was feasible and safe in treating high-risk neuroblastoma, resulting in 3-year overall survival and event-free survival of 60% and 36%, respectively [14].

Neuroblastomas, like many solid tumors, harbor a complex immunosuppressive microenvironment hindering immune-mediated tumor clearance. Tumor-infiltrating lymphocytes are often inactivated or exhausted due to immunosuppressive factors, including cytokines (IL-6, IL-10, TGF-β, galectin-1) secreted by tumor, stromal, and myeloid immune cells [15–19]. Furthermore, MYCN overexpression in neuroblastoma cells diminishes NKG2D ligands, impeding NK cell activation [20]. Gangliosides like GD2 (cell-bound or soluble) suppress immunity by binding to myeloid checkpoint Siglec-7 [21], and CD8^+^ T cell cytotoxicity is curtailed via intracellular granule interference [22]. Lymphocyte activation is further hampered by recruitment and induction of immunosuppressive cells, such as regulatory T cells (Tregs), tumor-associated macrophages (TAMs) and myeloid-derived suppressor cells (MDSCs) [23].

While often implicated in immunosuppression, myeloid cell subsets also possess potent anti-tumorigenic properties. This review comprehensively outlines the dualistic roles of myeloid cells in neuroblastoma. The first part summarizes their pro- and anti-tumorigenic functions during the development and progression of neuroblastoma. The second part discusses the involvement of myeloid cells in current treatment regimen and explores novel strategies for their targeting, including: (1) engaging myeloid cells as effectors, (2) ablating myeloid cells, (3) blocking recruitment to the TME and (4) reprogramming of myeloid cells.

## Myeloid subpopulations in the neuroblastoma microenvironment

**Fig. 1:**
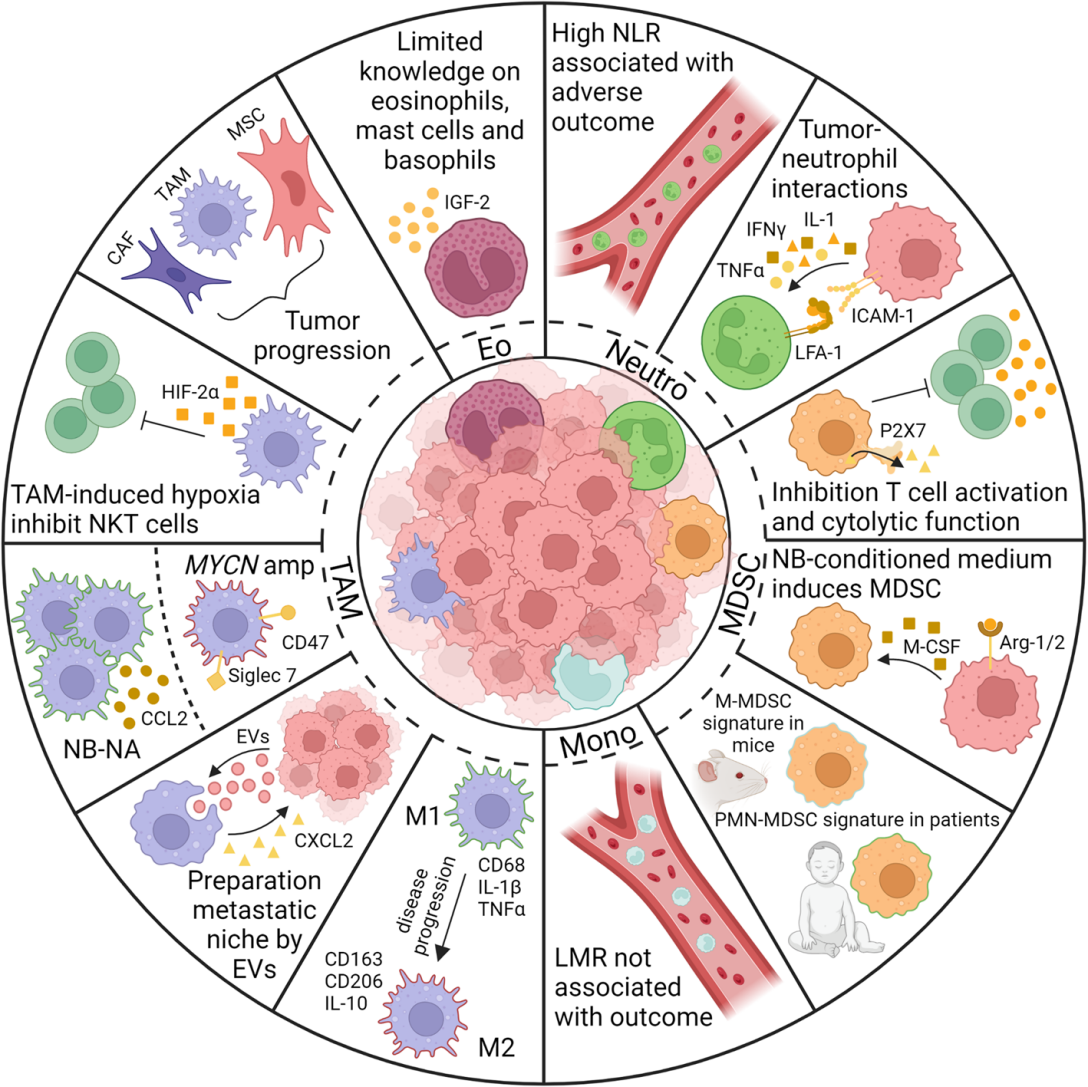
Overview of the interactions of myeloid cell subsets in the tumor microenvironment of neuroblastoma. A neuroblastoma tumor is shown in the center, surrounded by various myeloid immune cells: neutrophils, MDSC, monocytes, TAM, and eosinophils. For each of these cell types, pertinent interactions within the TME are depicted. For neutrophils, a high NLR is associated with unfavorable clinical outcomes. Reported tumor-neutrophil interactions involve the induction of neutrophil adhesion to neuroblastoma cell lines through IFNγ and IL-1, resulting in increased ICAM-1 expression on neutrophils. MDSCs are linked to T cell activation inhibition, notably through P2X7 receptor activation leading to increased ATP levels within the TME. Additionally, neuroblastoma-conditioned medium induces MDSCs through M-CSF and Arg-1/2. While most mouse MDSCs exhibit an M-MDSC phenotype, human MDSCs present a PMN-MDSC signature. For monocytes, LMR was not correlated with clinical outcomes in neuroblastoma patients. In neuroblastoma, TAMs undergo polarization from M1 to M2 as the disease progresses. Additionally, TAMs contribute to the preparation of the metastatic niche by taking up tumor-secreted EVs, resulting in the upregulation of immunosuppressive cytokines and genes associated with tumor cell extravasation, and via the CXCL2/CXCR2 axis. In *MYCN*-amplified neuroblastoma, TAMs exhibit elevated expression of macrophage-related immune checkpoints CD47 and Siglec7, in contrast to *MYCN*-nonamplified tumors. *MYCN*-nonamplified neuroblastoma is characterized by elevated CCL2 secretion by TAMs, resulting in the recruitment of TAMs, myeloid cells, and plasmacytoid DC. Furthermore, TAM-induced hypoxia leads to the inhibition of NK cells through HIF-2α production. TAMs also collaborate with CAFs and MSCs to promote tumor progression. Although our understanding of eosinophils, mast cells, and basophils is limited, IGF-2 secreted by eosinophils is suggested to play a role in neuroblastoma tumor growth. Abbreviations: Arg1/2 = arginase-1/2, ATP = adenosine triphosphate, CAF = cancer-associated fibroblast, CAF = cancer-associated fibroblasts, CCL2 = C–C motif chemokine ligand 2, CXCL2 = C-X-C motif chemokine ligand 2, CXCR2 = C-X-C motif chemokine receptor 2, DC = dendritic cell, Eo = eosinophil, EV = extracellular vesicles, EVs = extracellular vesicles, HIF-2a = hypoxia inducible factor 2α, ICAM-1 = intercellular adhesion molecule-1, IFNγ = interferon gamma, IGF-2 = insulin-like growth factor 2, LMR = lymphocyte-to-monocyte ratio, M-CSF = macrophage-colony stimulating factor, M-MDSC = monocytic myeloid-derived suppressor cells, MDSC = myeloid-derived suppressor cell, Mono = monocyte, MSC = mesenchymal stromal cell, MSC = mesenchymal stromal cells, NB = neuroblastoma, NB-NA = MYCN non-amplified neuroblastoma, Neutro = neutrophil, NK = natural killer, NLR = neutrophil-to-lymphocyte ratio, PMN-MDSC = polymorphonuclear myeloid-derived suppressor cells, TAM = tumor-associated macrophage, TME = tumor microenvironment

While the field of tumor immunology initially focused on dissecting the role of lymphocytes, there is a growing awareness of the significance of myeloid cells. Myeloid cells comprise monocytes, macrophages, granulocytes (mainly neutrophils and eosinophils), MDSC and certain DC subsets. Though some DCs are of myeloid origin, they are beyond the scope of this review, as they have been extensively covered elsewhere (reviewed in [24]). MDSC can be categorized into three subsets: polymorphonuclear MDSC (PMN-MDSC, CD33^+^CD14^−^CD15^+^LOX1^+^), monocytic MDSC (M-MDSC, CD33^+^CD14^+^CD15^−^HLA-DR^−/low^) and early MDSCs (E-MDSC, CD11b^+^CD33^+^CD14^−^CD15^−^) [25–27]. Identifying specific MDSC subsets can be challenging due to overlapping lineage markers with other immune cells such as neutrophils and monocytes (reviewed in [28]). However, the main defining characteristic of MDSCs is their immunosuppressive function. Recent markers such as LOX-1 and CD84 expression on PMN-MDSCs [26, 29] or gradient centrifugation [30] can aid in distinguishing PMN-MDSCs from neutrophils, as PMN-MDSCs reside in the low-density (peripheral blood mononuclear cells; PBMC) fraction, while neutrophils are high-density cells. Furthermore, M-MDSCs can be differentiated from monocytes by lower or absent HLA class II expression and increased C-X-C motif chemokine receptor 1 (CXCR1) expression [25, 31]. The challenges in accurately identifying MDSCs often lead to inconsistencies in nomenclature across studies. In this review, we adhere to the nomenclature used in the referenced literature, considering the mentioned immune cell subsets as they are described. Now, we will first discuss the involvement of each of these myeloid immune subsets in neuroblastoma, starting with neutrophils (Fig. 1).

### Neutrophils

Neutrophils are short-lived phagocytes (though their exact lifespan is heavily under debate) and are the most abundant leukocyte population in the human body, primarily residing in the bone marrow [32, 33]. Approximately 10^11^ neutrophils are released from the bone marrow and enter circulation daily, with an additional release during inflammatory conditions, upon which they migrate towards the sites of inflammation [34].

The prognostic significance of tumor-infiltrating neutrophils or the neutrophil-to-lymphocyte ratio (NLR) in various cancer types typically indicates unfavorable outcomes [35]. However, in the case of neuroblastoma, conflicting and inconclusive findings have been reported regarding the correlation between neutrophils and disease progression. Initial studies by Morandi et al. demonstrated increased neutrophil counts in peripheral blood samples from patients with localized neuroblastoma compared to patients with metastasized neuroblastoma, hence neutrophils were associated with improved overall survival (OS) [36]. Similarly, Zeng et al. found a positive correlation between higher numbers of tumor-infiltrating macrophages and neutrophils and increased OS [37]. Conversely, other studies did not observe significant associations between neutrophils and survival [38], or even found the opposite [39–41]. Zhang et al. reported that a higher NLR was associated with reduced OS [39]. Erbe et al. did not find a significant correlation between NLR and survival, but they did note that a higher neutrophils count was associated with shortened event-free survival (EFS) [41]. Lee et al. observed that elevated neutrophil counts were associated with a higher cumulative incidence of disease progression, although not significantly impacting EFS or OS [40]. However, in a subgroup analysis of high-risk neuroblastoma patients, the correlation was stronger, with a high neutrophil count significantly associated with reduced EFS. Although these results appear conflicting, there are indications that high neutrophils counts in blood may indicate a positive prognosis in localized neuroblastoma (low-risk), whereas high neutrophil counts in high-risk neuroblastoma are associated with a negative prognosis, as suggested by the studies of Morandi and Lee. Nevertheless, further investigations, specifically differentiating between low- and high-risk patients, are required to confirm these hypotheses in the future.

The interaction between neutrophils and neuroblastoma cells was studied already decades ago, albeit mostly in vitro. Two studies showed that cytokines like tumor necrosis factor alpha (TNFα), Interleukin (IL)-1 and interferon gamma (IFNγ) induce neutrophil adhesion to SK-N-SH and LAN-1 neuroblastoma cell lines by upregulating intercellular adhesion molecule-1 (ICAM-1) expression [42, 43]. Blocking lymphocyte function-associated antigen 1 (LFA-1, formed by CD11a and CD18), the ligand of ICAM-1, prevented cytokine-induced neutrophil adhesion to neuroblastoma cells. However, neutrophil binding to SK-N-MC cells was very high and independent of stimulatory cytokines or LFA-1, indicating multiple adhesion mechanisms exist for different neuroblastoma cell lines. Interestingly, co-culture experiments demonstrated that endothelial cells down-regulated adhesion receptor CD33 upon interaction with neuroblastoma cells, thus impairing neutrophil adhesion and extravasation [44]. Nevertheless, Fultang et al. indicated the presence of CD15^+^ neutrophils in neuroblastoma tumors, particularly in high-risk disease, suggesting their ability to extravasate and localize around the vasculature [45]. Direct co-culture of neuroblastoma cell lines with neutrophils exhibited both pro-tumorigenic (SMS-KCN and SMS-LHN) and anti-tumorigenic (LAN-1) effects, in a cell–cell contact independent manner [46]. These studies highlight both pro-tumorigenic and anti-tumorigenic effects of neutrophils and touch upon the intricate interplay between neutrophils and the neuroblastoma cells in the TME.

More recently, Martínez-Sanz and colleagues identified upregulated neutrophil-related mRNA transcripts (including *CGR3B*, *FPR1*, *S100A8/9*, and *SIGLEC9)* in both early and late-stage neuroblastoma, compared to healthy adrenal gland tissue [47]. However, the exact interactions between neutrophils and tumor cells in the TME remain largely unknown. After the initial interest in neutrophils, immunotherapy research focused more on harnessing the adaptive immune system and engaging NK cells through antibody therapy in the early 2000s, which may explain the scarcity of data on neutrophils. Furthermore, a nomenclature change has led to (immunosuppressive) neutrophils being described more frequently as PMN-MDSC in cancer. Consequently, we will now delve into the role of these cells in neuroblastoma.

### Myeloid-derived suppressor cells

MDSCs represent an immature immunosuppressive subset of myeloid cells that undergo expansion during pathological conditions such as inflammation and cancer [48, 49]. Their presence has been associated with poor clinical outcomes in various cancer types [50]. Under the influence of continuous cytokine production within the TME, undifferentiated myeloid cells from the bone marrow enter circulation and infiltrate the tumor, where they often fail to mature (reviewed in [50–52]). MDSCs exert immunosuppressive effects by suppressing T and NK cell activation and cytotoxicity, while promoting Treg and M2 macrophage polarization. They employ various mechanisms to induce immune suppression, including the production of reactive oxygen species (ROS), nitric oxide (NO), anti-inflammatory cytokines, and the depletion of L-arginine via arginase (Arg)-1 [48, 53, 54]. In general, M-MDSCs exhibit higher immunosuppressive activity than PMN-MDSCs, suppressing both antigen-specific and non-specific T cell responses [48].

In neuroblastoma-bearing mice, both PMN-MDSC and M-MDSC induce T cell suppression [55–59]. Notably, TH-MYCN mice, which exhibit spontaneous neuroblastoma development due to MYCN overexpression, showed a higher PMN-MDSC abundance compared to M-MDSC [60]. Conversely, in a commonly used neuroblastoma mouse model involving A/J mice injected with NXS2 neuroblastoma cells intravenously, M-MDSC displayed higher levels of Arg-1 and iNOS, and produced higher levels of TGF-β1 and ROS compared to PMN-MDSC [56]. This hints at M-MDSCs' superior suppressive potency compared to PMN-MDSC, a trend observed in other cancer types as well [61, 62]. Co-injection of M-MDSCs with NXS2 neuroblastoma cells promoted tumor growth more than PMN-MDSC co-injection, underscoring their distinct roles. MDSCs' immunosuppressive actions involved P2X7 receptor activation, which elevates extracellular adenosine triphosphate (ATP) levels within the TME. P2X7R agonist BzATP induced M-MDSCs to secrete chemoattractant C–C motif chemokine ligand (CCL)-2, and upregulated Arg-1, ROS, and TGF-β1 in MDSC cell lines MSC-1 and MSC-2.

Furthermore, neuroblastoma-conditioned medium has been shown to induce MDSCs. Cultivating hematopoietic progenitors or monocytes in such media generated immunosuppressive myeloid cells [57, 59, 63]. Intriguingly, Arg-2-expressing neuroblastoma cells depleted local and circulating arginine levels, similar to the mechanism employed by MDSCs [63]. Arg-1 in neuroblastoma hindered myeloid activation and suppressed CD34^+^ bone marrow progenitor cell proliferation, suggesting that arginine deprivation contributes to MDSC induction in neuroblastoma [64]. Additionally, the macrophage-colony stimulating factor (M-CSF)/colony stimulating factor 1 receptor (CSF-1R) axis was suggested to drive myeloid differentiation into M-MDSCs in neuroblastoma [57]. Elevated M-CSF, CSF-1R, CD68, and CD14 correlated with poor patient outcomes. Intriguingly, primary human monocytes polarized into suppressive CD14^+^CSF-1R^+^ M-MDSC upon co-culture with neuroblastoma cells, mediated by tumor-originating M-CSF and attenuated through CSF-1R blockade.

Limited neuroblastoma patient data exists on MDSC subsets in circulation. Santilli et al. found elevated PMN-MDSCs in the peripheral blood of patients with neuroblastoma vs. controls [65]. Furthermore, high-risk neuroblastoma exhibited higher levels of CD33^+^CD11b^+^HLA-DR^−^ MDSCs (M-MDSC) than low-risk neuroblastoma [60]. Unexpectedly, responsive high-risk patients had more circulating HLA-DR^−^ MDSCs compared to patients who were refractory to therapy [66]. Recent single-cell transcriptomics unveiled exclusive tumor-infiltrated immature myeloid/neutrophil cells in bone marrow metastasis of neuroblastoma patients, suggesting PMN-MDSC involvement [64]. PMN-MDSC signatures were observed in primary neuroblastoma tumors based on single-cell transcriptomic analysis [58], with higher proportions of MDSCs detected in *MYCN*-amplified tumors compared to *MYCN*-nonamplified tumors. At relapse, tumors had increased MDSC proportions compared to diagnosis [60].

In summary, in vivo studies implicate a more important role for M-MDSC in neuroblastoma tumor progression compared to PMN-MDSC due to their prominent suppressive function. However, it remains uncertain if this translates to the human context. Furthermore, it is important to note that MDSC spatial distribution, abundance, and interactions with other immune cell subsets within the TME remain undisclosed.

### Monocytes & macrophages

Monocytes and macrophages play a significant role within the myeloid compartment of the TME and possess phagocytic abilities. Unlike neutrophils, circulating monocyte levels or leukocyte-to-monocyte ratio (LMR) are not linked to overall survival [38, 41]. Tumor-derived chemokines and cytokines can recruit monocytes to the TME, where they can differentiate into monocyte-derived macrophages [67]. Additionally, tissue-resident macrophages can also be found in the TME, originating from yolk-sac precursors and exhibiting self-renewal capabilities [68]. Furthermore, recruited M-MDSCs can differentiate into macrophages at the tumor site [69]. Macrophages are traditionally categorized into M1 (pro-inflammatory) and M2 (anti-inflammatory) states [70], yet they exhibit notable plasticity and heterogeneity along this spectrum.

Macrophages in human neuroblastoma have a predominant M2-like phenotype, with some M1 presence [45, 58, 71–74]. In the TH-MYCN mouse model, TAMs transitioned from an M1 phenotype (MHCII^high^/CD206^low^) to an M2 phenotype (MHCII^low^/CD206^high^) during tumor progression [55]. Additionally, the number of infiltrating immune cells and the proportion of TAMs (F4/80^+^/CD45^+^) among total infiltrating cells increased in advanced TH-MYCN tumors. In vitro, neuroblastoma-exposed monocytes/macrophages upregulated M2 markers (CD163, CD204, IL-10) [73, 75]. Conversely, Fultang et al. observed that neuroblastoma cells polarized macrophages toward an M1 phenotype (CD68^+^CD163^−^) [45]. Consistent with these findings, low numbers of M2-polarized macrophages were observed to be correlated with unfavorable clinical outcome in neuroblastoma [37, 76]. Importantly, TAM emerge as the predominant PD-L1-expressing cells within the TMA, which was associated with improved clinical outcome in high-risk patients [77]. Finally, single-cell RNA sequencing revealed an augmented presence of an inflammatory monocyte cell state (*IL1B*^high^*S100A*^high^) in neuroblastoma compared to normal tissue [78].

Macrophages drive neuroblastoma dissemination by creating premetastatic niches, facilitating tumor cell colonization [79]. Studies utilizing SK-N-BE-derived primary tumors revealed the release of extracellular vesicles that are engulfed by macrophages at distant sites such as the liver and bone marrow. Consequently, this led to upregulation of immunosuppressive cytokines (IL-10), as well as genes involved in the recruitment of MDSC and tumor cell extravasation, rendering the premetastatic niche more attractive for tumor cell colonization. In accordance, high numbers of M2 macrophages in the primary neuroblastoma tumor correlated with the presence of bone marrow metastasis and poor clinical outcome [71, 73, 80]. Moreover, macrophage-derived CXCL2 promoted neuroblastoma invasiveness, but CXCL2/CXCR2 mechanism in this context remains unclear [73]. These are the only studies investigating the role of myeloid cells in neuroblastoma metastasis, though this has been described more extensively in other cancers, indicating there is a knowledge gap on this topic. However, recently it was described that macrophage migration inhibitory factor (MIF) and midkine (MDK) are expressed in neuroblastoma [81, 82], factors that are known to be involved in metastasis in other cancers [83, 84]. Unraveling the involvement of MIF and MDK in neuroblastoma metastasis could provide important information in the future.

*MYCN*-amplified and non-amplified neuroblastomas exhibit varying macrophage infiltration, reflecting distinct immune profiles due to specific genetic alterations. Chromosome 11q deletion or *MYCN* amplification in neuroblastomas exhibit higher numbers of M2-polarized (CD163^+^) macrophages and an activated Th2-lymphocytes/M2-macrophage axis compared to neuroblastoma lacking these mutations [72, 73, 85]. Interestingly, *MYCN* amplification is generally associated with lower immune infiltrate than non-amplified cases [86]. Theruvath et al. confirmed this by re-analyzing public databases, revealing higher Siglec 7 and CD47 expression on *MYCN*-amplified neuroblastoma macrophages [21]. In metastatic non-amplified neuroblastomas, TAM infiltration was higher compared to locoregional tumors, with a predominant M2 phenotype [71]. Furthermore, metastatic non-amplified tumors in patients ≥ 18 months showed elevated inflammation-related gene expression compared to those < 18 months. Consistent with these findings, a study involving 129 NA-NB patients reported upregulation expression of CD14, IL-6 and TGF-β in patients with poor clinical outcome [87].

The NB-tag mouse model [88], resembling human non-amplified neuroblastoma, recruits TAMs, myeloid cells, and plasmacytoid DCs through CCL2 signaling [88, 89]. Macrophage-secreted CCL2 is prominent in non-amplified tumors, and inversely correlated with *MYCN* amplification [87, 90]. The recruited DCs subsequently attracted CD4^+^ and CD8^+^ T cells via CCL22 and CCL19 [89]. In vitro and in vivo studies have demonstrated that CCL2-attracted TAMs in neuroblastoma enhance IL-6 expression, promote tumor growth and inhibit apoptosis [87, 88]. Furthermore, TAMs in non-amplified tumors activate STAT3 in neuroblastoma cells, upregulating c-Myc that reciprocally induces CCL2 secretion, forming a positive feedback loop [88]. Consistent with these findings, a recent in vivo study reported that depletion of macrophages expressing the CCL2 receptor (CCR2) prevented tumor formation in an ALK-mutated TH-MYCN mouse model [91].

Hypoxia, common in cancer due to insufficient oxygen supply (reviewed in [92]), upregulates membrane-bound TNFα on neuroblastoma cells, triggering TAMs to produce CCL20 [93]. This TAM-derived CCL20 recruits natural killer T (NKT) which eliminate pro-tumorigenic CD1d^+^ TAM at the tumor site [87]. Despite this anti-tumorigenic potential, hypoxia impairs NKT cell function against CD1d^+^ TAMs [93]. Additionally, hypoxia inducible factor 2α (HIF-2α)-producing TAMs were detected alongside neuroblastoma neural crest-like cells in the perivascular niche, where high levels of vascular endothelial growth factor (VEGF) were observed [94]. This suggests a cooperative interaction between neuroblastoma crest-like cells and macrophages to promote angiogenesis via HIF‐2α-mediated VEGF expression.

In neuroblastoma, TAMs collaborate with cancer-associated fibroblasts (CAFs) and mesenchymal stromal cells (MSC) to support tumor progression, especially in relapse patients. TAMs induce CAF proliferation [73], and MSCs/CAFs shield monocytes from apoptosis through in an IL-6-dependent manner [95]. The interactions of monocytes and MSC resulted in the significant upregulation of several pro-tumorigenic cytokines and chemokines, including TGF-β1, MCP-1, IL-6, IL-8, and IL-4. Furthermore, the abundance of MSC and CAFs was correlated with tumor progression, including high histological malignancy and low infiltration of T and NK cells [73, 96].

In summary, despite some conflicting findings, most studies suggest a notable role for M2-polarized TAMs in neuroblastoma progression, driving c-Myc expression, CAF recruitment, and angiogenesis.

### Eosinophils

Currently, limited knowledge exists regarding the roles of granulocytes other than neutrophils in neuroblastoma, including eosinophils, basophils, and mast cells. To our knowledge, no literature is available discussing the role of basophils and mast cells in neuroblastoma. Eosinophils, usually associated with allergic reactions and immune responses against parasites [97], may have a minor role in neuroblastoma. In cancer, eosinophils generally display anti-tumorigenic properties, although some pro-tumorigenic effects have also been described (reviewed in [98]).

Eosinophils were first identified in neuroblastoma in the 1990s when a study of 21 tumors revealed their presence, along with insulin-like growth factor 2 (IGF-2) expression in these cells [99]. The authors proposed a mechanism whereby IGF-2, expressed both autocrinally and paracrinally (including by eosinophils), promotes tumor growth, but this mechanism was never confirmed. A subsequent study reported a positive link between low eosinophil count and patient survival [100]. Though not yet reported in neuroblastoma, tumor-infiltrating eosinophils are common in various cancer types [98], suggesting they are possibly present in the neuroblastoma TME as well.

### The role of epigenetics in myeloid cells and therapy resistance

Myeloid cells exhibit a high degree of plasticity, characterized by their ability to deviate from typical differentiation pathways and generate diverse tolerogenic myeloid cell states within the TME. A substantial component of this plasticity can be attributed to epigenetic mechanisms, with myeloid cells displaying marked responsiveness to histone modifications, alterations in DNA methylation patterns, chromatin remodeling, and the regulatory impact of non-coding RNA molecules [101–103]. Importantly, abnormal epigenetic modifications have been reported in the development and progression of cancer and therapy resistance [104, 105], and are closely related to altered glucose, lipid, and amino acid metabolism in the TME as thoroughly reviewed elsewhere [103, 105, 106]. For instance, histone citrullination, catalyzed by peptide arginine deiminase (PAD), is associated with the creation of neutrophil extracellular traps (NETs) in cancer, contributing to both innate immunity and tumor progression [107]. In addition, Hypoxia and M1 macrophages drive histone lactylation through lactate accumulation in the TME. This upregulated methyltransferase-like 3 (METTL3) in infiltrating myeloid cells in colon cancer via H3K18 lactylation, crucial for the transcription of immunosuppressive genes [108]. Furthermore, reduction of methylation at the Arg1 and STAT3 promoter regions has been reported in ex vivo induced MDSCs, resulting in the release of STAT3-associated cytokines IL-6 and IL-10, which subsequently activated STAT3 phosphorylation. The phosphorylated STAT3, in turn, bound to the Arg1 and S100A8 promoter regions, leading to upregulation of Arg1 and S100A8, thereby augmenting the immunosuppressive capacity of MDSCs [109, 110]. Fetahu et al. identified key transcription factors in myeloid cells of neuroblastoma bone marrow metastases linked to open chromatin regions in genes associated with M2 polarization, tumor growth, and metastases, including *IL-10*, *TIMP1*, and *EREG* [111]. Finally, several studies suggest that PD-1 and PD-L1 expression in tumors is decreased due to epigenetic changes, resulting in resistance to immune checkpoint therapy. Interestingly, the DNA-demethylating agent azacytidine increased PD-L1 expression in non-small cell lung cancer patients [112, 113].

In neuroblastoma, two different epigenetic states have been defined: mesenchymal or neuro crest cell-like cells (MES) with a less-differentiated state, and adrenergic or sympathetic noradrenergic cells (ADRN) with a more differentiated state [114–116]. In primary neuroblastomas, the initial tumor mainly consists of the ADRN state [114, 115], but the MES state becomes more prominent during relapse and metastasis [117]. Studies showed that human neuroblastoma cells with stronger MES signature have a higher basal inflammatory state, promote T cell infiltration by secreting inflammatory cytokines, and respond to immune checkpoint therapy in an immunocompetent mouse model [118, 119]. Specifically, *PRRX1* a component of the mesenchymal core regulatory circuitry, activates the transcription of MHC class I and APP genes which subsequently enhance the immunogenic state of neuroblastoma cells [119]. Furthermore, sensing of double-stranded RNA in MES state neuroblastoma cell lines resulted in secretion of proinflammatory cytokines, enrichment of inflammatory transcriptomic signatures, and increased tumor killing by T cells in vitro [118]. Interestingly, genetically switching unresponsive ADRN state neuroblastoma cell lines towards the MES state fully restored responsiveness. These studies did, however, not investigate the role of myeloid cells in relation to the epigenetic states of neuroblastoma cells.

In summary, epigenetic alterations are important for immunotherapy resistance, governing not just the expression of immune checkpoint inhibitors, but also influencing immune cell infiltration, antigen presentation, and the expression and release of cytokine profiles within the tumor. Furthermore, they drive myeloid cell reprogramming toward immunosuppressive subsets within the TME, amplifying their suppressive potential.

## The role of myeloid cells in immunotherapy

**Fig. 2:**
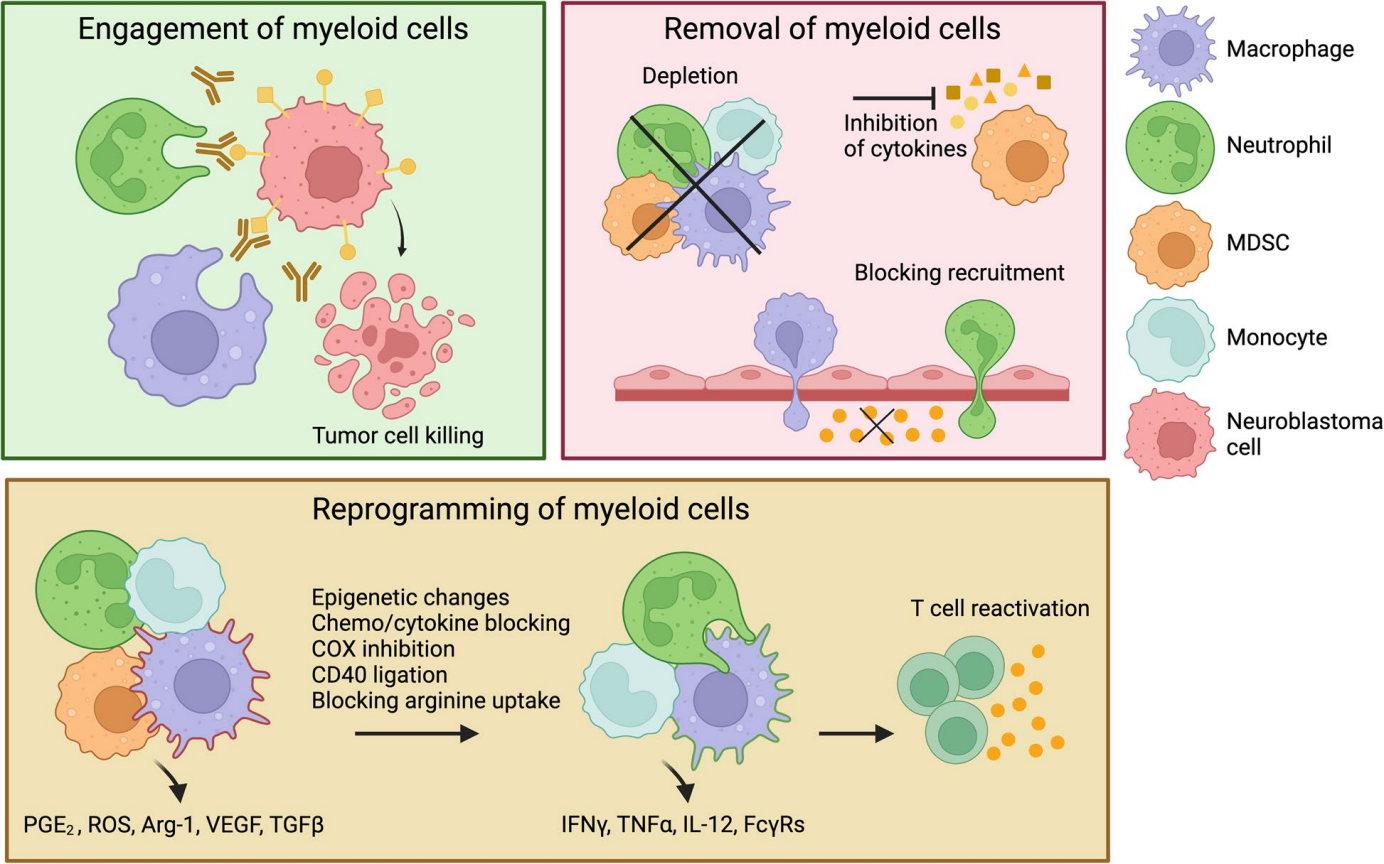
Three main strategies of targeting myeloid cells in neuroblastoma. Myeloid cells can be 1.) engaged as effector cells of immunotherapy via ADCC. 2.) removed from the TME by depletion with antibodies (anti-CD11b, anti-CD105, anti-CD33, or anti-CSF-1R) or myoablative chemotherapy. Additionally, myeloid cells can be depleted via inhibition of cytokines via IL6R blockade, or inhibition of TGFβ or STAT3. Finally, their recruitment can be blocked with CCR2 inhibitors, or anti-CCL2 or anti- TGFβ. 3.) reprogrammed to an anti-tumor, immunostimulatory phenotype, leading to reactivation of T cells. Myeloid cell reprogramming can be achieved by inducing epigenetic modifications, inhibiting cytokines and chemokines, suppressing COX activity, stimulating CD40 ligation, and preventing arginine uptake. Abbreviations: ADCC = antigen-dependent cellular cytotoxicity, Arg-1 = arginase-1, CCL2 = C–C motif chemokine ligand 2, CCR2 = CCL2 receptor, COX = cyclooxygenase, CSF-1R = colony stimulating factor 1 receptor, FcγRs = Fc gamma receptors, IFNγ = interferon gamma, ROS = reactive oxygen species, TGFβ = transforming growth factor beta, TME = tumor microenvironment, TNFα = tumor necrosis factor alpha, VEGF = vascular endothelial growth factor

As previously discussed, myeloid cells have been shown to predominantly exhibit a pro-tumorigenic role in neuroblastoma, making them attractive targets for immunotherapeutic interventions. In this section, we provide a summary of established and emerging therapeutic strategies that specifically target myeloid cells in neuroblastoma (Fig. 2).

### Myeloid cells as effector cells of immunotherapy

#### Myeloid effector cells in anti-GD2 immunotherapy

It is widely recognized that in addition to NK cells, macrophages possess the potential to serve as effector cells in current anti-GD2 antibody therapy. As early as 1990, it was demonstrated that M-CSF-differentiated macrophages could phagocytose neuroblastoma tumor cells upon stimulation with the 3F8 anti-GD2 antibody [120]. Recent studies have further emphasized the efficacy of macrophages as effector cells in antibody immunotherapy [21, 77, 121]. However, within the TME, macrophages (or TAMs) often exhibit inactivation, refractoriness, or highly immunosuppressive traits, thereby contributing to antibody therapy resistance. Interestingly, in two neuroblastoma patient cohorts, the presence of TAMs expressing PD-L1, as well as the expression of the SLAMF7 pathway and CD163 in TAMs, was found to be beneficial for high-risk patients and correlated with improved survival following immunotherapy [77]. While this may appear counterintuitive, it suggests that despite their immunosuppressive characteristics, TAMs can still be engaged as effector cells in immunotherapy.

Similarly, despite their association with tumor progression in high-risk neuroblastoma, neutrophils and monocytes (or MDSCs) can still be effectively engaged in anti-GD2 antibody therapy (Table 1) [46]. In the late 1980s and early 1990s, it was demonstrated that neutrophils could perform antigen-dependent cellular cytotoxicity (ADCC) against human neuroblastoma cells in vitro with anti-GD2 antibodies (ch14.18 or 3F8 IgG) [122–125]. Remarkably, Barker and colleagues discovered that granulocytes, displayed superior killing capacity against NMB-7 neuroblastoma cells compared to PBMC [124], which is generally the opposite in IgG antibody therapy. Furthermore, neutrophils derived from neuroblastoma patients (stage IV) retained potent ex vivo killing ability against neuroblastoma cells, matching that of neutrophils from healthy donors. This neutrophil-mediated killing is marked by the release of lytic granules [125] and dependent on CD11b/CD18 (collectively known as membrane-activated complex 1 (Mac-1) or complement receptor 3 (CR3)), as well as FcγRII and FcγRIII [126].

**Table 1:** Comprehensive overview of treatments in neuroblastoma engaging myeloid cells as effector cells

Therapeutic target	Treatment	Studied population	(Pre-)clinical model	Outcome	Ref
GD2	Anti-GD2	PMN	In vitro, ex vivo	Induces ADCC of NB cell lines by healthy and patient-derived PMN, via FcγRII, FcγRIII and Mac-1	[122–127]
GD2	Anti-GD2	PMN, other myeloid cells	Clinical trials	PMN-mediated ADCC via FcγRII and FcγRIII, improvement EFS and OS	[12, 13, 77, 128–130]
GD2	Anti-GD2 + GM-CSF	PMN, other myeloid cells	In vitro, clinical trials	GM-CSF increases and activates myeloid cells, increases CD11b expression, enhances PMN-mediated ADCC by anti-GD2	[12, 123, 125, 126, 128, 131, 132]
GD2	Anti-GD2 + G-CSF	PMN	In vitro	Similar to GM-CSF, enhances PMN-mediated ADCC by anti-GD2	[133]
GD2	Anti-GD2 + retinoic acid	PMN	In vitro	Retinoic acid attracts PMN via IL-8 production	[134]
IL-2R	IL-2 or IL-2-expressing NB cells	Eosinophils	In patients, clinical trials	Increase of eosinophil numbers, elevated IL-5 serum levels	[131, 135–137]
GD2	IgA anti-GD2	PMN, TAM	In vitro, 9464D syngeneic and IMR32 xenograft models	Superior neutrophil activation compared to IgG anti-GD2 especially with patient-derived PMN, prolonged survival in vivo	[138, 139]
GD2 + CD47	Anti-GD2 IgG + anti-CD47	PMN, TAM	In vitro, multiple mouse models	Enhanced phagocytosis and PMN-mediated killing of NB cells by disrupting CD47/SIRPα axis, prolonged survival in vivo, influx of intratumoral macrophages	[21, 47, 121]
B7-H3	B7-H3/GD2 bispecific or anti-B7-H3 ADC	Myeloid cells	PDX models	Efficacy possibly mediated by myeloid cells	[140, 141]
GPC2	anti-GPC2 ADC	Monocytes, TAM, MDSC	NXS2 xenograft and 9464D syngeneic models	Recruitment monocytes, TAM and MDSC, phagocytosis of NB cells, prolonged survival	[142, 143]

Subsequently, in two clinical trials investigating 3F8 anti-GD2 antibody therapy, it was demonstrated that neutrophil-mediated ADCC was indeed responsible for therapeutic efficacy in neuroblastoma patients [128, 129]. Activated neutrophils, characterized by the CD11b activation epitope CBRM1/5, were associated with prolonged progression-free survival [129]. Moreover, an *FCGR2A* polymorphism in patients showed a correlation with clinical outcome following anti-GD2 antibody and GM-CSF therapy [130, 144]. The FcγRIIa-R131 variant exhibited higher affinity for the murine IgG3 3F8 antibody than FcγRIIa-H131, resulting in enhanced ADCC. Consequently, patients homozygous for the *FCGR2A-R131* allele demonstrated extended progression-free survival after treatment with murine 3F8 antibody and GM-CSF compared to patients who were heterozygous or homozygous for *FCGR2A-H131* [130]. It is noteworthy that the affinity of these polymorphisms is reversed for *human* IgG1 antibodies, thus leading to an opposite correlation with dinutuximab (anti-GD2, CH14.18 antibody), which is of the human IgG1 isotype [144]. Given the high expression of FcγRIIa on neutrophils, macrophages, and monocytes and its absence on NK cells, the prognostic value of this FcγRIIa polymorphism indicates that myeloid cells constitute a major effector cell population in antibody therapy for neuroblastoma.

Although the introduction of anti-GD2 antibody therapy significantly improved survival rates, resistance still develops in many patients. In vitro studies have revealed that GD2 expression levels do not correlate with neutrophil-mediated ADCC, suggesting abundant GD2 levels on all neuroblastoma cells [127]. However, the effectiveness of neutrophil-mediated tumor cell killing was diminished in tumor cells displaying high rates of antibody internalization, thus proposing a potential mechanism for treatment resistance.

Previously, anti-GD2 immunotherapy was combined with IL-2. However, this was discontinued, since a recent randomized phase III trial of patients with high-risk neuroblastoma showed that the addition of IL-2 to anti-GD2 antibody therapy did not improve outcome and increased treatment toxicity [8]. Besides its stimulatory effects on NK-cell mediated ADCC [145], IL-2 was shown to upregulate expression of the adhesion molecule CD18 on neutrophils, but to decrease total neutrophil numbers [131, 146]. Additionally, two immune monitoring studies in neuroblastoma observed that IL-2 cycles increased eosinophil counts [131, 147]. It is generally known that eosinophils can be pre-activated by IL-2 and subsequently exert ADCC [148]. However, eosinophils are likely not involved in tumor killing, since similar levels of ADCC were found in patients with high compared to low eosinophil count and eosinophil counts were not significantly correlated with survival. Furthermore, long-term infusion of anti-GD2 in combination with IL-2 resulted in induction of Tregs, which inversely correlated with IFN-y levels and progression-free survival [135].

In the United States, anti-GD2 antibody therapy is enhanced with GM-CSF, but its accessibility and availability outside the country are restricted due to regulatory constraints. GM-CSF increases numbers of circulating neutrophils, monocytes, and eosinophils and promotes the release of myeloid cell-associated factors such as CXCL11, CCL17, CCL23, and MCP4 [131]. However, the primary mechanism of GM-CSF is augmenting neutrophil-mediated ADCC and to a lesser extent activating macrophages and monocytes. Both in vitro and in patients, the combination of GM-CSF with anti-GD2 antibodies activates neutrophils and upregulates CD11b expression [12, 123, 125, 126, 128]. Particularly in patients with refractory or minimal residual disease, the addition of GM-CSF to the treatment regimen has shown favorable responses [12, 132]. Interestingly, the route of GM-CSF administration influences the degree of neutrophil activation, with subcutaneous injection resulting in a higher percentage of activated neutrophils compared to intravenous injection [129]. However, in other cancer types, such as liver carcinoma and glioblastoma, GM-CSF is involved in the induction of MDSCs [149, 150], but during immunotherapy in neuroblastoma, GM-CSF appears to activate neutrophils instead. This may be attributed to the concurrent antibody therapy skewing neutrophils toward an anti-tumor phenotype and/or the intermittent cycles of GM-CSF administration rather than a continuous treatment. It is plausible that low-concentration, chronic GM-CSF stimulation induces MDSCs through negative feedback loops, whereas high-concentration, acute GM-CSF stimulation as applied in neuroblastoma therapy, activates neutrophils. Since GM-CSF is not available in Europe, several research groups in the Netherlands have explored the potential use of G-CSF as an alternative to GM-CSF [133]. They concluded that G-CSF exhibited comparable potency to GM-CSF in enhancing ADCC by neutrophils from both healthy donors and neuroblastoma patients. Similar to GM-CSF-enhanced ADCC, the killing mechanism was dependent on FcγRIIa and CD11b/CD18. Given the similarity in performance between G-CSF and GM-CSF, the authors proposed that the addition of G-CSF to anti-GD2 immunotherapy should be evaluated in patients.

Next to GM-CSF, the current treatment regimen for high-risk neuroblastoma includes 13-*cis* retinoic acid, also known as isotretinoin. In vitro studies have demonstrated that retinoic acid inhibits neuroblastoma cell growth and promotes cellular differentiation [151]. While retinoic acid alone does not improve overall survival [152], pretreatment with retinoic acid enhances the susceptibility of neuroblastoma cells to antibody therapy. Furthermore, differentiated neuroblastoma cells treated with retinoic acid produce IL-8, a crucial cytokine involved in the attraction of neutrophils [134]. IL-8-mediated neutrophil attraction may augment neutrophil-mediated killing induced by anti-GD2 antibody therapy. However, studies conducted in other advanced cancers have reported that IL-8 production and subsequent recruitment of neutrophils/PMN-MDSC can be detrimental, particularly in the context of checkpoint inhibitor treatment [153, 154]. Additionally, retinoic acid can exhibit both immunosuppressive effects, such as differentiation of immunosuppressive TAM [155, 156], and immunostimulatory effects, such as induction of inflammatory anti-tumor macrophages in other cancer types [157, 158]. It is important to note though that most of these studies involved an isomer of 13-*cis* retinoic acid—all-trans retinoic acid—which may have a slightly different mechanism of action. These studies combined underscore the potential effects of 13-*cis* retinoic acid treatment on the TME, although its specific impact on the neuroblastoma TME remains largely unknown.

#### Improving myeloid engagement in anti-GD2 immunotherapy

Several strategies have been developed to improve efficacy of anti-GD2 antibody therapy by targeting myeloid cells. For example, neutrophils exhibit stronger activation in response to IgA antibodies, via their Fc alpha receptor (FcαRI or CD89), compared to IgG antibodies. Our laboratory demonstrated the superior ability of IgA1 anti-GD2 antibodies, which share the same variable region as dinutuximab (ch14.18), in mediating neutrophil-mediated tumor cell killing compared to IgG1 ch14.18 [138]. Importantly, IgA anti-GD2 antibodies did not induce neuropathic pain in mice, unlike IgG anti-GD2 antibodies, primarily due to the absence of the complement factor C1q-binding site. Consequently, IgA antibodies do not activate complement on GD2 expressing nerves, a factor implicated in IgG-induced neuropathic pain [159]. Moreover, we developed a novel form of IgA anti-GD2 antibody (IgA3.0 ch14.18) that is suitable for clinical application, lacking *O*-glycosylation and featuring mutations that enhance antibody stability and prolong half-life [139]. In long-term mouse models, including xenograft and immunocompetent models with IMR32 and 9464D cells, IgA3.0 ch14.18 demonstrated remarkable anti-tumor efficacy. Furthermore, IgA3.0 ch14.18 effectively induced tumor cell killing by patient neutrophils, while IgG1 ch14.18-mediated tumor cell killing was significantly diminished with patient PBMC compared to healthy donor PBMC. In summary, harnessing and enhancing the anti-tumorigenic activity of neutrophils holds significant promise as a novel approach in the treatment of neuroblastoma.

Another novel strategy to enhance anti-GD2 antibody therapy is to block the CD47/SIRPα axis, analogous to immune checkpoints in T cell biology such as CTLA-4 and PD1/PD-L1. CD47, known as the 'don't eat me' signal, is frequently upregulated in various cancers, including neuroblastoma [47]. Through its interaction with the SIRPα (signal regulatory protein alpha or CD172a) receptor, CD47 inhibits myeloid cell-mediated killing. In the context of neuroblastoma, Theruvath et al. demonstrated that the combination of IgG anti-GD2 and anti-CD47 therapy enhanced macrophage-mediated phagocytosis of neuroblastoma cells in vitro synergistically and led to tumor eradication in mouse xenograft and syngeneic models [21]. The efficacy of this combination therapy primarily relied on phagocytosis of tumor cells by macrophages. Furthermore, the study revealed that ligation of anti-GD2 antibodies upregulated surface calreticulin, which primes macrophages for phagocytosis and disrupts the interaction between GD2 and Siglec 7, another myeloid checkpoint. Consequently, the authors found that the synergy was specific to the combination of anti-GD2 antibodies, as anti-CD47 therapy showed less synergy with antibodies targeting other antigens, such as B7-H3. Encouraged by these findings, a clinical trial investigating the combination of anti-GD2 (dinutuximab) and anti-CD47 (magrolimab) antibodies in neuroblastoma relapse patients was initiated (NCT04751383).

Similarly, anti-CD47 synergizes with antibodies solely recognizing the *O*-acetyl variant of GD2, developed by the lab of Stephane Birklé [121]. Interestingly, anti-*O*-acetyl-GD2 antibody therapy enhanced CD47 expression on tumor cells and induced influx of F4/80^+^ macrophages in a NXS2 liver metastasis model, which further explains the synergy of anti-GD2 and anti-CD47 therapy. Though the previous studies did not find a significant role for neutrophils in mouse models upon anti-GD2 and anti-CD47 combination treatment, Martínez-Sanz and colleagues showed that human neutrophils can be unleashed by this combination strategy in vitro [47]. Dinutuximab induced neutrophil-mediated killing of unmodified neuroblastoma cell lines up to 5–20%, whereas up to 80% killing was achieved in CD47 knockout cell lines. It will be interesting to evaluate in patients whether only macrophages or also other myeloid cells, such as neutrophils are involved in tumor clearance upon combination therapy.

Recently, in other cancers it was described that neutrophils can be activated to their full killing potential by supplementing IgG antibody therapy with TNF and CD40 agonists [160]. Since Voeller and colleagues already described synergy between CD40 agonists and anti-GD2 therapy in neuroblastoma [161], the addition of TNF and CD40 agonists to the treatment regimen could prove a promising new strategy.

#### Novel strategies for antibody immunotherapy engaging myeloid cells

Currently, alternative targets for antibody therapy in neuroblastoma are being explored, to circumvent the problem of GD2 downregulation, which is particularly prominent in a mesenchymal subset of neuroblastoma cells. Studies have identified B7-H3 as a viable alternative target, given its sustained expression in mesenchymal neuroblastoma cells [162, 163]. The laboratory of Paul Sondel is currently investigating a bispecific SNIPER antibody that targets both GD2 and B7-H3, demonstrating superior efficacy compared to monospecific anti-B7-H3 antibodies and lacking the induction of neuropathic pain [140]. Furthermore, Kendsersky et al. have demonstrated in vivo efficacy of the B7-H3-targeting antibody–drug conjugate (ADC) m276-SL-PBD in patient-derived xenograft (PDX) models of neuroblastoma [141]. However, the involvement of myeloid cells in the effector mechanisms of these novel therapies remains unknown.

Another promising target against which ADCs have been developed is the oncoprotein GPC2, which is overexpressed in neuroblastoma and drives tumor cell proliferation [142, 143]. Anti-GPC2 ADCs have been shown to induce immunogenic cell death in mouse models (NXS2 and 9464D) with GPC2 overexpression, leading to the recruitment of monocytes, macrophages, and MDSCs to the TME. Alongside T cells, macrophages have emerged as major mediators of the anti-GPC2 ADC treatment, and the combination of anti-GPC2 ADCs with anti-CD47 blockade has demonstrated a modest reduction in tumor burden [143].

Though myeloid cells have a negative image in the context of cancer due to their immunosuppressive traits, their potential as effector cells should not be underestimated. Though neutrophils and macrophages are already important mediators in antibody therapy their full potential is not yet reached and myeloid cell engagement can be enhanced, for example by targeting the CD47/SIRPα myeloid checkpoint.

### Removal of suppressive myeloid cells from the TME

#### Direct depletion of myeloid cells

A potential strategy to counteract the suppressive function of myeloid cells in the TME is the depletion of these cell populations as summarized in Table 2. One approach involves using anti-CD11b antibodies to target and deplete all cells of the myeloid lineage. In a study conducted on NXS2 tumor-bearing mice treated with dinutuximab, anti-CD11b antibodies resulted in a modest delay in tumor growth and prolonged survival [164]. Another approach employs anti-CD105 antibodies, which specifically target a transmembrane co-receptor for both TGF-β and bone morphogenic protein-9 (BMP-9). By administering anti-CD105 antibodies, not only monocytes but also MDS and endothelial cells could be depleted [165]. This approach resulted in improved efficacy of anti-GD2 antibodies when combined with adoptively transferred activated human NK cells in neuroblastoma patient-derived xenograft (PDX) models.

**Table 2:** Comprehensive overview of treatments in neuroblastoma removing myeloid cells from the tumor microenvironment

Therapeutic target	Treatment	Treatment strategy	Studied population	Pre-clinical model	Outcome	Ref
CD11b	Anti-CD11b	Depletion	Myeloid lineage	NXS2 xenograft model	Tumor growth delay, prolonged survival	[164]
CD105	Anti-CD105	Depletion	Monocytes, MSC, endothelial cells	PDX models	Improved efficacy of anti-GD2 antibodies when combined with adoptively transferred activated human NK cells	[165]
Ly6G	Anti-Ly6G (+ GD2-EATs)	Depletion	PMN, PMN-MDSC	PDX models	Higher T cell and M-MDSC infiltration in the tumor, prolonged survival	[166]
Ly6C	Anti-Ly6C (+ GD2-EATs)	Depletion	Monocytes, M-MDSC	PDX models	Decrease in TAM and increase in intratumoral PMN-MDSC and T cells, prolonged survival	[166]
Macrophages/CSF1R	Clodronate liposomes or anti-CSF-1R (+ GD2-EATs)	Depletion	TAM	PDX models	Decrease in M-MDSC and increase in PMN-MDSC and T cells, prolonged survival	[166]
CSF1R	Inhibitor BLZ945 + anti-CSF-1R	Depletion	Monocytes, TAM	CHLA-136 and CHLA-255 xenograft and PDX models	Improvement of chemotherapeutic efficacy, which was independent of T cell contribution	[167]
CSF-1	Small interfering RNAs	Depletion	TAM	SK-N-AS and SK-N-DZ xenograft models	Decreased intratumoral TAM, matrix metalloprotease 12 levels and angiogenesis, suppression of tumor outgrowth	[168]
Glucocorticoid receptor	Dexamethason (+ GD2-EATs)	Depletion and reprogramming	Monocytes, TAM, M-MDSC	PDX models	Decrease in IL-2, IL-6, and TNF-α release, increase in PMN-MDSC and T cells, and enhanced survival	[166]
CD33	Gemtuzumab ozogamicin	Depletion	MDSC	In vitro	Restored T cell proliferation, MDSC cell death, enhanced anti-GD2 CAR-T cell activity	[169]
CD1d/GM-CSF	NKT cells	Depletion and reprogramming	MDSC, TAM	In vitro, CHLA-255 xenograft model	Killing of suppressive TAM and MDSC via CD1d, NKT cells-derived GM-CSF differentiates TAM to M1, decrease of IL-10 expression in PMN-MDSC	[87, 170–172]
Apoptosis (via ROS)	Doxorubicin	Depletion and reprogramming	MDSC	In vitro, Neuro2a syngeneic model	Increased T cell proliferation and function, reduction in Tregs, less suppressive myeloid cells, inhibition of TAM polarization to M2, improved efficacy of anti-GD2 and adoptive T cell transfer, improved survival	[173, 174]
Apoptosis (via ROS)	Doxorubicin	Depletion	MDSC	In vitro	improved antigen-specific CTL-killing, via upregulating CD3ζ and L-selectin	[175]
Forming DNA adducts	Cisplatin	Depletion	M-MDSC	SK-N-DX xenograft model	Reducing tumor burden	[176]
Thymidylate synthase	5-FU	Depletion	MDSC	Syngeneic mouse model	Reduction of CD11b + cells in the tumor, improved efficacy of anti-GD2	[164]
Thymidylate synthase	5-FU	Depletion	MDSC	Neuro2A-bearing chimeras	Improved local tumor growth-inhibitory effect of recipient leukocyte infusion	[177]
STAT3	AZD1480, ruxolitinib	Inhibition of cytokines	TAM	NBT2 model in NB-TAG and NSG mice	Reduction of TAM-mediated upregulation of MYCN tumor growth inhibition	[88]
STAT3	STAT3 inhibition, or knockdown	Inhibition of cytokines	TAM	In vitro	Abrogates drug-resistance to etoposide and melphalan due to monocyte-derived IL-6 inducing STAT3 signaling	[178]
IL-6R	Tocilizumab	Inhibition of cytokines	TAM	CHLA-255 and CHLA-136 xenograft models	Apoptosis of TAM	[95]
TGF-βR1	Galunisertib	Inhibition of cytokines	TAM, MSC	CHLA-255 and CHLA-136 xenograft models	Decrease of IL-6 production by NB cells, apoptosis of TAM, restored NK cell activity	[95]
TGF-β	Anti-TGF-β	Blocking recruitment	TAM	TH-MYCN mice	Decreased recruitment of M2 TAM	[179]
COX enzymes	Aspirin	Blocking recruitment	MDSC, immature DC, TAM	TH-MYCN mice	Decreased recruitment of MDSCs, immature DCs and TAMs, reduced tumor burden	[55, 180]
CXCR2	Anti-CXCR2	Blocking recruitment	TAM, CAF	In vitro	Abrogation of the invasive ability of NB cells induced by TAM-derived CXCL2	[73]
CCL2	S1P2 agonist AB1 and JTE-013	Blocking recruitment	TAM	SK-N-AS xenograft model	Inhibition of TAM infiltration, reduced VEGF expression and reduced tumor outgrowth	[181, 182]
DNA synthesis	Oxaliplatin	Blocking recruitment	PMN	TH-MYCN mice	Low-dose decreases recruitment PMN	[179]

Park et al. studied myeloid depletion methods in a neuroblastoma PDX model with bispecific GD2 antibodies (GD2-EATs) [166]. They employed various approaches including anti-Ly6G antibodies (targeting neutrophils and PMN-MDSC), anti-Ly6C antibodies (targeting M-MDSC and TAM), anti-CSF-1R antibodies or clodronate liposome (targeting macrophages) and dexamethasone (targeting monocytes). All improved T cell infiltration and survival when combined with GD2-EATs, with macrophage depletion being most effective. Similarly, depletion of human monocytes and macrophages through CSF-1R inhibition using BLZ945, in combination with anti-CSF-1R treatment, enhanced chemotherapeutic efficacy in immunodeficient NOD/SCID mice with neuroblastoma xenografts, independently of T cell contribution [167]. Additionally, Abraham et al. demonstrated that intratumoral injections of small interfering RNAs targeting mouse CSF-1 resulted in significant suppression of tumor growth in SK-N-AS and SK-N-DZ neuroblastoma xenografts, accompanied by decreased TAM infiltration [168]. Finally, treatment of human MDSC with the anti-CD33 ADC gemtuzumab ozogamicin led to cell death in vitro [169]. In co-culture experiments, gemtuzumab ozogamicin restored T cell proliferation and enhanced the activity of anti-GD2 CAR-T cells.

Importantly, depletion of myeloid cells is considered a ‘sledgehammer approach’ as myeloid effector cells that are required for immunotherapy are depleted as well. Targeting specific suppressive myeloid subsets might be more effective. NKT cells offer promise, as they can selectively eliminate immunosuppressive TAMs and MDSCs via CD1d interaction [87, 170]. NKT cells also produce GM-CSF, inducing M1-like TAMs [87, 171] and decreased IL-10 expression in PMN-MDSCs [170], thereby reverting their suppressive function. Although NKT cells are typically present in low numbers within the TME, GD2-CAR NKT cells have shown promise by maintaining their cytotoxic activity against suppressive TAMs [172]. Therefore, harnessing GD2-CAR NKT cells could serve as an indirect approach to deplete immunosuppressive TAMs and MDSCs in the TME.

#### Myeloablative chemotherapy

The chemotherapeutic doxorubicin (DOX) is currently the most specific drug for the selective removal of MDSC through ROS-mediated apoptosis induction [183], enhancing T cell activity and reducing Tregs in a BALB/c NB mouse model [173, 174]. Residual myeloid cells exhibited reduced expression of Arg-1, indoleamine-pyrrole 2,3-dioxygenase (IDO), as well as STAT3, STAT5, and STAT6, which are key signaling pathways involved in MDSC activation. Notably, in contrast to findings in a murine study on breast cancer [183], DOX affected macrophages in neuroblastoma by inhibiting their polarization from M1 to M2 phenotype [174]. Dopamine also yielded similar outcomes, albeit less potent than DOX. Furthermore, DOX enhanced antigen-specific CD8^+^ T cell cytotoxicity against neuroblastoma cells by upregulating CD3ζ and L-selectin [175]. Importantly, DOX improved efficacy of anti-GD2 therapy and adoptive T cell transfer, resulting in enhanced survival rates in BALB/c mice [173].

Platinum-based chemotherapeutics, such as cisplatin, have been demonstrated to inhibit STAT signaling, as evidenced by their ability to suppress cyclooxygenase (COX)-2-expressing M-MDSCs induced by melanoma tumor cells in vitro [184]. This inhibitory effect of cisplatin on M-MDSCs was further observed in patients with head and neck squamous cell carcinoma who underwent intravenous cisplatin treatment, leading to a drastic reduction in M-MDSC frequency. Furthermore, the surviving M-MDSCs exhibited decreased expression of COX2 and Arg-1, a result of attenuated STAT3 signaling, consequently impairing their ex vivo T cell inhibitory capacity. In a mouse model of SK-N-DX neuroblastoma, cisplatin was effective in reducing tumor burden. However, the specific mechanism underlying its action in this model was not investigated [176]. 5-FU, a chemotherapeutic agent, exerts its effects by targeting thymidylate synthase and depleting myeloid suppressive cells. In a syngeneic neuroblastoma mouse model, treatment with 5-FU resulted in a reduction of CD11b^+^ cells within the tumor and enhanced the efficacy of anti-GD2 antibodies [164]. Furthermore, depletion of MDSCs following 5-FU treatment improved the inhibitory effect of recipient leukocyte infusion on local tumor growth in murine neuroblastoma (Neuro2A)-bearing chimeras [177].

Importantly, it should be noted that one of the current chemotherapy regimens COJEC, has been associated with the induction of M2 macrophages in *MYCN*-amplified neuroblastoma [185]. Similarly, a neuroblastoma PDX model demonstrated that macrophage infiltration promoted the outgrowth of tumor cells that had survived COJEC-like chemotherapy. Consequently, the COJEC regimen could contribute to the development of resistance to anti-GD2 therapy in neuroblastoma and T cell-based therapeutics. Currently, the COJEC regimen, consisting of cisplatin, vincristine, carboplatin, etoposide, and cyclophosphamide, is frequently applied [186]. From a clinical point of view there is a strong wish to add anti-GD2 therapy during induction chemotherapy. Therefore, it could be important to explore alternative chemotherapeutic regimens that can synergize with immunotherapy.

#### Inhibition of cytokines

As described above, IL-6 stimulation and STAT signaling in myeloid cells are associated with immunosuppressive characteristics, including Arg-1 expression. STAT3 inhibition with AZD1480 or ruxolitinib reduced TAM-mediated MYC upregulation, restraining NBT2 tumor growth in NB-tag and NSG mice [88]. Moreover, Ara et al. demonstrated that IL-6 from monocytes activated STAT3 in neuroblastoma cells, causing drug resistance against etoposide and melphalan in vitro, which was counteracted by STAT3 inhibition or knockdown [178]. Studies in other cancer types suggest that STAT3 inhibition impedes the local proliferation of TAMs, thereby reducing their abundance within the tumor [187]. Furthermore, Louault et al. showed that IL-6 produced by MSC and neuroblastoma cells promoted the survival of TAMs ex vivo [95]. Apoptosis of TAMs was observed upon blocking the IL-6 receptor using the monoclonal antibody Tocilizumab. Upstream, TGF-β stimulated IL-6 production in neuroblastoma cells and MSC, contributing to the suppression of NK cell cytotoxic activity. Treatment with Galunisertib, a TGF-βR1 inhibitor, decreased IL-6 production in co-cultures and restored the activity of NK cells. Therefore, targeting the IL-6/TGF-β axis holds promise as a strategy to selectively deplete TAMs from the neuroblastoma TME.

#### Blocking recruitment of myeloid cells

Rather than eradicating the myeloid compartment entirely, an alternative strategy involves impeding myeloid cell recruitment to the tumor site. Overexpressed COX enzymes in neuroblastoma drive myeloid cell attraction by synthesizing prostaglandins, including PGE_2_ [55, 188, 189]. Blocking COX with aspirin in TH-MYCN mice led to diminished tumor burden and reduced tumor-associated myeloid cells like MDSCs, immature DCs, and TAMs [55, 180].

The CCL2/CCR2 axis is pivotal for recruiting monocytes, myeloid cells, and plasmacytoid DCs in *MYCN*-nonamplified neuroblastoma [89]. In other cancer types, inhibiting the CCL2/CCR2 axis with CCR2 inhibitors or anti-CCL2 antibodies has demonstrated reduced myeloid cell recruitment and improved clinical outcomes [190–192]. Sphingosine-1 (S1P), a bioactive lipid, induced CCL2 expression in neuroblastoma via S1P2 [181]. Inhibiting S1P2 with the stable derivative AB1, but not its precursor JTE-013, decreased macrophage infiltration in neuroblastoma xenografts, underscoring AB1's potential to inhibit TAM infiltration [182].

Furthermore, certain chemotherapeutic and immunotherapeutic drugs impact immune cell recruitment to tumors. For example, anti-TGF-β reduced M2 TAM recruitment in a neuroblastoma model, and low-dose oxaliplatin hindered neutrophil migration to tumors [179].

While depleting or blocking myeloid cells has been proven effective in neuroblastoma, it also depletes potential immunotherapy effectors. Additionally, targeting specific cytokines or immunosuppressive mediators produced by MDSC can be useful, but this only targets a small part of the problem. Reprogramming suppressive myeloid cells may be the optimal strategy, interrupting immunosuppressive traits while preserving myeloid cells as effectors of immunotherapy.

### Reprogramming suppressive myeloid cells into an active phenotype

Finally, a highly elegant approach to target immunosuppressive myeloid cells involves reprogramming their suppressive phenotype into an antitumor, immunostimulatory state (Table 3). Various published therapeutic approaches have demonstrated successful myeloid cell reprogramming with consequent T cell activation. For instance, ibrutinib, an irreversible molecular inhibitor of Bruton's tyrosine kinase (BTK), was shown to reverse T cell suppression mediated by murine MDSC in a neuroblastoma mouse model [193]. This was evidenced by altered NO production and decreased mRNA expression of immunosuppressive factors *Ido*, *Arg*, and *Tgfb*. Moreover, ibrutinib-mediated BTK inhibition increased CD8^+^ T cell infiltration and enhanced response to anti-PD-L1 checkpoint inhibitor therapy. As previously discussed, neuroblastoma-derived factors hinder early myeloid cell differentiation to monocytes and macrophages, promoting monocyte suppressive function via M-CSF/CSF-1R interaction [57]. CSF-1R inhibitor BLZ945 effectively blocked CSF-1R-expressing suppressive myeloid cell generation and reversed tumor-educated monocyte suppression. Combining CSF-1R inhibition with anti-PD-1/PD-L1 immune checkpoint blockade in TH-MYCN mice enhanced activation of tumor-reactive T cells. Interestingly, PD-1 blockade induced enhanced T cell M-CSF secretion, enhancing monocyte suppressive capacity [194]. Thus, BLZ945-CSF-1R inhibition combined with PD-1 blockade synergistically controlled tumor growth. Additionally, catechins such as Polyphenon E exhibit neuroblastoma anticancer effects by inhibiting MDSC activity. Administered via drinking water in multiple neuroblastoma mouse models, Polyphenon E hindered MDSC development and mobility, and facilitated their differentiation into neutrophils through 67 kDa laminin receptor signaling and G-CSF induction [65]. Moreover, it lowered Arg-1 expression on MDSCs, promoting T cell proliferation in patient metastases. Another treatment strategy that potentiates T cells by reprogramming myeloid cells is addition of neutrophil-activating protein (NAP) in therapies like CAR T cell or oncolytic virus treatments. Tumino et al*.* reported an increase in circulating PMN-MDSC numbers upon GD2.CAR T-cell therapy [195]. Circulating PMN-MDSCs inversely correlated with GD2.CAR T-cell levels, potentially predicting treatment response. Moreover, Stroncek et al. demonstrated that monocytes inhibit the expansion of GD2.CAR T cells in neuroblastoma patients [196]. In accordance with this, GD2.CAR T cells were ineffective in a syngeneic NXS2 neuroblastoma model. However, GD2.CAR T cells designed to express NAP delayed tumor outgrowth by generating a ‘hot’ TME with high infiltration of neutrophils, M1 macrophages, NK cells, CD8^+^ T cells, DCs, and a reduced number of Tregs [197], which potentiated GD2.CAR T cell therapy. Likewise, oncolytic virus therapy can be enhanced by incorporating NAP into the virus load. Oncolytic Vaccinia viruses carrying a GD2 mimotope were ineffective against NXS2 tumors, but the inclusion of NAP led to tumor growth inhibition [198].

**Table 3:** Comprehensive overview of treatments in neuroblastoma reprogramming myeloid cells into an immunostimulatory phenotype

Therapeutic target	Treatment	Studied population	Pre-clinical model	Outcome	Ref
BTK	Ibrutinib	MDSC	9464D syngeneic model	Decrease of NO expression and of *Ido*, *Arg*, and *Tgfb* mRNA expression in MDSC, reversion of T cell suppression, increased CD8^+^ T cell infiltration, improved response to anti-PD-L1	[193]
CSF1R (and PD1)	BLZ945 (+ anti-PD-1)	MDSC, monocytes	TH-MYCN mice	Reverted suppressive functions of tumor-educated monocytes, abolished induction of MDSC, activation of tumor-reactive T cells, improved anti-PD-1/PD-L1 blockade	[57, 194]
TLR2	NAP (+ CAR T cells or oncolytic virus)	PMN	NXS2 syngeneic model	infiltration of neutrophils, M1 macrophages, NK cells, CD8^+^ T cells and DCs, reduced number of Tregs, reduced tumor growth	[197, 198]
mPGES-1	Small molecule inhibitor	CAFs, TAM	SK-N-AS xenograft model and TH-MYCN mice	PGE_2_ production was inhibited by CAFs, resulting in reduced tumor growth, impaired angiogenesis, and polarization of TAM to pro-inflammatory M1	[199]
Histone deacetylation	Vorinostat (+ retinoic acid or MIBG)	TAM, M-MDSC	TH-MYCN mice, phase I clinical trial	Upregulation of GD2 in NB cells, increase of TAM with mixed M1 and M2 phenotype, depletion of M-MDSC, downregulation of suppressive markers on myeloid cells, beneficial response rates in NB patients	[200–202]
IFNγ	IFNγ-expressing MSC	TAM	CHLA-255 orthotopic xenograft model	TAM polarization to M1 phenotype, reduced tumor growth, prolonged survival	[203]
FABP4	Anti-IL-1a	TAM	In vitro	Suppression of FABP4-mediated migration and invasion of NB cells	[204]
CD40	CD40 agonist	TAM	NXS2 syngeneic model	M1 polarization of TAMs	[205]
CD40	CD40 agonist + CpG	TAM	NXS2 syngeneic model	Anti-CD40 primes TAM to respond to CpG, resulting in M1 polarization and tumor growth inhibition	[206]
CD40	CD40 agonist + CpG + chemo, or radiation, anti-GD2 and anti-CTLA-4	TAM	9464D syngeneic model	Synergistic anti-tumor effects of combination treatments via repolarization of TAMs to M1 phenotype	[161, 207]
Rac2	Rac2 knockout	TAM	Rac2^−/−^ mice with 9464D model	Rac2 knockout promotes M1 TAM differentiation, reduced tumor growth	[208]
67 kDa laminin receptor	Polyphenon E	MDSC, PMN	TH-MYCN mice	Impairment of development and motility of MDSC, MDSC differentiation towards PMN, induction of G-CSF, decreased Arg-1 expression on remaining MDSC, increased T cell proliferation	[65]
BRD3/4	BET bromodomain inhibitors	Myeloid cells	In vitro, multiple in vivo models	Tumor growth inhibition, *MYCN* downregulation, possibly reprogramming of myeloid cells	[209–211]

Other studies did report strategies to reprogram myeloid cells, but did not observe or investigate the indirect effects on T cells. COX inhibitors have shown promise in limiting myeloid infiltration by inhibiting PGE_2_, but their use is constrained by potential side effects. An alternative approach by Kock et al. selectively targeted microsomal prostaglandin E synthase-1 (mPGES-1) using a small molecule inhibitor in an immunocompetent transgenic neuroblastoma mouse model with *MYCN* oncogene expression [199]. This targeted inhibition of mPGES-1 suppressed PGE_2_ production specifically in CAFs, resulting in reduced tumor growth, impaired angiogenesis, and macrophage polarization towards a pro-inflammatory M1 phenotype. In addition, human FABP4-expressing macrophages were discovered to promote migration, invasion, and tumor growth of neuroblastoma cell lines [204]. This was facilitated through FABP4 binding to ATPB, which triggered ATPB ubiquitination, reduced ATP levels, and deactivated the NF-kB/RelA-IL-1a pathways, driving macrophages into an M2 phenotype. Blocking antibodies against IL-1a effectively countered FABP4-induced increased migration and invasion. Ligating CD40 offers another approach to target myeloid cells, as it activates antigen-presenting cells including macrophages. In murine NXS2 models, anti-CD40 treatment led to delayed tumor progression by inducing a proinflammatory M1 phenotype in macrophages and stimulating Th1 cytokine production [205]. This treatment also synergized with CpG-containing oligodeoxynucleotides (CpG), promoting upregulation of intracellular Toll-like receptor (TLR)-9 and generating NO, IFNγ, TNFα, and IL-12 in A/J mice bearing NXS2 tumors [206]. When combined with multidrug chemotherapy, radiation, anti-GD2, and anti-CTLA-4, this anti-CD40 and CpG therapy exhibited potent anti-tumor effects in mice with 9464D neuroblastoma tumors, repolarizing TAMs towards an M1 phenotype [161]. Furthermore, histone deacetylase (HDAC) inhibitors have been explored in cancer therapy due to their ability to prevent histone deacetylation, resulting in altered gene and protein expression [212]. In neuroblastoma, the HDAC inhibitor vorinostat has shown enhanced efficacy in combination with anti-GD2 antibody treatment in a TH-MYCN mouse model [200]. Vorinostat treatment increased GD2 expression in neuroblastoma tumors, improving responsiveness to anti-GD2 therapy. It also modified the myeloid composition of the TME, enhancing TAMs with mixed M1 and M2 phenotypes while reducing M-MDSC. Vorinostat-treated TAMs exhibited heightened ADCC capability, downregulated immunosuppressive genes, and upregulated FcyRI and FcyRII/III. Although clinical trials are lacking, the combination of anti-GD2 therapy and vorinostat holds promise for neuroblastoma treatment. Vorinostat was well-tolerated in a phase I trial with isotretinoin [201] and showed favorable response rates in a phase II trial with 131I-metaiodobenzylguanidine (MIBG) [202].

Furthermore, several therapeutic strategies have been reported that suggest alteration of gene expression of tumor cells or macrophages as a promising strategy to reprogram myeloid cells. BET bromodomain inhibitors like JQ1 and I-BET726 displace BRD4 from the *MYCN* promoter, leading to improved survival in various in vivo neuroblastoma models by inhibiting tumor growth and *MYCN* downregulation [209, 210]. Combined PI3K and BRD4 inhibition also shows promise, suppressing *MYCN* expression and inhibiting neuroblastoma cell growth and metastasis both in vitro and in vivo [211]. While not investigated in neuroblastoma, PI3K/BRD4 blockade has demonstrated immunomodulatory effects. In tumor-bearing mice, it reduces MDSC recruitment, enhances MHCII^+^ TAMs with a pro-inflammatory M1 phenotype, and promotes CD8^+^ T cell infiltration and activation within the tumor [213]. These findings suggest that aside from *MYCN*, PI3K/BRD4 blockade may influence myeloid cell migration and polarization, potentially contributing to its anti-tumor effects in neuroblastoma. Further research is needed to fully understand this mechanism. In addition, Rac2, a small GTPase, has been linked to the polarization of macrophages from M1 to M2 phenotype in vivo [208]. Rac2-deficient mice studies indicated that although macrophage infiltration into the TME remained unchanged, the absence of Rac2 led to a prevalent M1 phenotype in macrophages. In a syngeneic neuroblastoma (9464D) model, Rac2-deficient mice exhibited significantly reduced tumor growth, suggesting the contribution of M2 macrophages to tumor progression. Notably, Rac2 is also implicated in tumor-induced angiogenesis, implying that the impact of Rac2 knockdown on tumor inhibition might involve both M1 macrophage polarization and the suppression of tumor angiogenesis. Potential therapeutic strategies involving Rac2 inhibition, using inhibitors like NSC23766, wortmannin, and LY294002, warrant further exploration in neuroblastoma cancer development [214, 215].

Finally, some studies have been published that demonstrate the possibility to target cytokines to reprogram myeloid cells. Relation et al. introduced a novel strategy involving MSCs engineered to express IFNγ, injected directly into neuroblastoma tumors [203]. This led to decreased tumor proliferation and improved survival in a CHL-255 orthotopic neuroblastoma model, without increasing macrophage infiltration, but instead polarizing TAMs to an M1 phenotype. Additionally, incubating neuroblastoma cell lines with human monocytes increased IL-1β and TNFα-expressing macrophages, signifying an M1 phenotype via AKT phosphorylation [45]. IL-1β and TNFα from these macrophages stimulated arginine metabolism in neuroblastoma cells, promoting tumor cell proliferation. AKT inhibition using MK-2206 halted this effect by blocking IL-1β and TNFα secretion by macrophages. Blocking arginine uptake in tumor cells with L-NAME or depleting arginine with BCT-100 hindered neuroblastoma cell differentiation. BCT-100 treatment delayed neuroblastoma development in a TH-MYCN mouse model, prolonging survival. A phase I/II study with BCT-100 treatment is currently conducted in patients with neuroblastoma (NCT03455140). To our knowledge, results of this study have not been published yet.

Reprogramming the myeloid compartment in neuroblastoma offers an elegant strategy, preserving potential effector cells. This approach can also indirectly reactivate tumor-infiltrating T cells, and enhance T cell therapies, such as CAR T cells and checkpoint inhibitors. In addition to all myeloid-targeting treatments discussed in this review, many other treatment strategies are promising, but have not yet been studied in neuroblastoma. Some examples are the inhibition of myeloperoxidase, preventing lipid peroxidation in PMN-MDSC, inhibition of fatty acid transport protein 2 (FATP2), preventing uptake of arachidonic acid and synthesis of PGE_2_, DR5 agonists inducing MDSC apoptosis via TNF-related apoptosis-inducing ligand (TRAIL) and inhibition of Arg-1 [216–219]. Strategies involving IDO, NO, or TOLLIP inhibition, ATRA treatment or other HDAC inhibitors than vorinostat, such as entinostat and ricolinostat were proven effective as well and are thoroughly reviewed elsewhere [28, 220–222]. These approaches hold opportunities for new neuroblastoma treatments, and should be further investigated in the future.

## Discussion

As in other solid tumors, the presence of neutrophils, MDSCs and macrophages in neuroblastoma is generally associated with worse survival and immunosuppression. Neutrophils appear to be associated with a worse prognosis in high-risk neuroblastoma, though exact mechanisms remain unknown and are obscured by changing nomenclature, with (suppressive) neutrophils now often referred to as PMN-MDSC. It is clear that MDSC are immunosuppressive in both patients and in mouse models. Interestingly, we observed that M-MDSC were more immunosuppressive than PMN-MDSC in mouse models, but due to limited patient studies it is uncertain whether this is the case in neuroblastoma patients as well. Regarding macrophages, research findings diverge. While some noted M2 macrophage induction by neuroblastoma, others primarily observed M1 macrophages, with both types potentially linked to poor clinical outcome. These discrepancies likely stem from distinct genetic profiles within neuroblastoma; tumors with chromosome 11q deletion or *MYCN* amplification tend to favor M2 phenotypes, whereas *MYCN*-nonamplified tumors have a higher prevalence of M1 macrophages [21, 72, 73, 85].

Because of their immunosuppressive nature and association with disease progression, myeloid cells are an excellent target for immunotherapy. Interestingly, immunosuppressive myeloid cells can still be engaged as effector cells, as is illustrated by the fact that neutrophils are important effector cells in anti-GD2 antibody therapy. This major role for neutrophils appears to be specific for anti-GD2 immunotherapy and/or neuroblastoma since neutrophils are much less involved as effectors of IgG antibody therapy in other cancers. One potential explanation for this phenomenon could be the timing of immunotherapy, often administered shortly after ASCT (90–120 days). Neutrophils, being highly abundant and among the first immune cells to recover post-ASCT, may possess a relative advantage compared to immune cells such as NK cells [223]. Additionally, post-ASCT NK cell recovery revealed a sustained enhanced metabolic immune cell profile relative to pre-ASCT levels in multiple myeloma patients [224, 225]. This is accompanied by a shift in NK cell subset distribution, correlating with reduced progression-free survival. These findings imply long-lasting ASCT-induced impacts on NK cell function.

In summary, substantial effort is put in developing T cell- and NKT cell-based therapeutics for neuroblastoma, among which CAR T cells and PD1/PD-L checkpoint inhibitors are the most prominent. However, results have been disappointing, apart from a breakthrough regarding GD2.CAR T cells for neuroblastoma patients with low tumor burden [14]. For adaptive immunity to be restored in neuroblastoma, first the immunosuppressive myeloid cells must be tackled. In this review, we highlighted several myeloid-targeting strategies that restored the ability of T cells to kill tumor cells, including depletion of myeloid cells using anti-Ly6G, anti-CD33 or the BTK inhibitor ibrutinib and myeloid reprogramming by DOX chemotherapy, anti-CSF1R, administration of Polyphenon E, or the addition of NAP to therapies. The combination of myeloid-targeting drugs and T cell-based therapeutics for neuroblastoma holds great promise for the future.

## Data Availability

This review article summarizes and discusses available literature and did not generate new data.

## Abbreviations

ADC: Antibody-drug conjugate
ADCC: Antigen-dependent cellular cytotoxicity
ADNR: Adrenergic noradrenergic cells
Arg-1: Arginase 1
ASC: Autologous stem cell transplantation
ATP: Adenosine triphosphate
BMP-9: Bone morphogenic protein-9
BTK: Bruton's tyrosine kinase
CAFs: Cancer-associated fibroblasts
CAR: Chimeric antigen receptor
CCL: C-C motif chemokine ligand
COX: Cyclooxygenase
CpG: CpG-containing oligodeoxynucleotides
CR3: Complement receptor 3
CSF-1R: Colony stimulating factor 1 receptor
CXCR: C-X-C motif chemokine receptor
DC: Dendritic cells
DOX: Doxorubicin
E-MDSC: Early myeloid-derived suppressor cells
EFS: Event-free survival
FcαRI: Fc alpha receptor
GD2: Disialoganglioside
GM-CSF: Granulocyte/macrophage-Colony Stimulating Factor
HDAC: Histone deacetylase
HIF-2α: Hypoxia inducible factor 2α
HLA: Human leukocyte antigen
ICAM-1: Intercellular adhesion molecule-1
IFNγ: Interferon gamma
IGF-2: Insulin-like growth factor 2
IL: Interleukin
L-NAME: N-nitro-L-arginine
LFA-1: Lymphocyte function-associated antigen 1
LMR: Leukocyte-to-monocyte ratio
M-CSF: Macrophage-colony stimulating factor
MDK: Midkine
MES: Mesenchymal cell-like cells
METTL: Methyltransferase-like 3
MIF: Macrophage migration inhibitory factor
M-MDSC: Monocytic myeloid-derived suppressor cells
Mac-1: Membrane-activated complex 1
MDSC: Myeloid-derived suppressor cells
mPGES-1: Prostaglandin E synthase-1
MSC: Mesenchymal stromal cells
NA-NB: MYCN non-amplified neuroblastomas
NAP: Neutrophil-activating protein
NET: Neutrophil extracellular traps
NK: Natural killer
NKT: Natural killer T
NLR: Neutrophil-to-lymphocyte ratio
NO: Nitric oxide
OS: Overall survival
PAD: Peptide arginine deiminase
PBMC: Peripheral blood mononuclear cells
PDX: Patient-derived xenograft
PMN: Polymorphonuclear cells
PMN-MDSC: Polymorphonuclear myeloid-derived suppressor cells
ROS: Reactive oxygen species
S1P: Lipid sphingosine-1
SIRPα: Signal regulatory protein alpha
TAMs: Tumor-associated macrophages
TGF-β: Transforming growth factor beta
TILs: Tumor-infiltrating lymphocytes
TLR: Toll-like receptor
TME: Tumor microenvironment
TNFα: Tumor necrosis factor alpha
VEGF: Vascular endothelial growth factor
